# Role of Akt Isoforms Controlling Cancer Stem Cell Survival, Phenotype and Self-Renewal

**DOI:** 10.3390/biomedicines6010029

**Published:** 2018-03-07

**Authors:** Sergio Rivas, Carla Gómez-Oro, Inés M. Antón, Francisco Wandosell

**Affiliations:** 1 Centro de Biología Molecular Severo Ochoa (CSIC-UAM), 28049 Madrid, Spain; srivas@cnb.csic.es; 2 Centro Nacional de Biotecnología (CNB-CSIC), 28049 Madrid, Spain; cgomez@cnb.csic.es; 3 Centro de Investigación Biomédica en Red de Enfermedades Neurodegenerativas (CIBERNED), 28031 Madrid, Spain

**Keywords:** signaling in cancer, Akt, PI3K, glioma, CSCs, TICs, proliferation, survival

## Abstract

The cancer stem cell (CSC) hypothesis suggests that tumours are maintained by a subpopulation of cells with stem cell properties. Although the existence of CSCs was initially described in human leukaemia, less evidence exists for CSCs in solid tumours. Recently, a CD133+ cell subpopulation was isolated from human brain tumours exhibiting stem cell properties in vitro as well as the capacity to initiate tumours in vivo. In the present work, we try to summarize the data showing that some elements of the Phosphoinositide 3-kinase Class I (PI3K)/ Thymoma viral oncogene protein kinase (Akt) pathway, such the activity of PI3K Class I or Akt2, are necessary to maintain the CSC-like phenotype as well as survival of CSCs (also denoted as tumour-initiating cells (TICs)). Our data and other laboratory data permit a working hypothesis in which each Akt isoform plays an important and specific role in CSC/TIC growth, self-renewal, maintaining survival, and epithelial-mesenchymal transition (EMT) phenotype, not only in breast cancer, but also in glioma. We suggest that a more complete understanding is needed of the possible roles of isoforms in human tumours (iso-signalling determination). Thus, a comprehensive analysis of how hierarchical signalling is assembled during oncogenesis, how cancer landmarks are interconnected to favour CSC and tumour growth, and how some protein isoforms play a specific role in CSCs to ensure that survival and proliferation must be done in order to propose/generate new therapeutic approaches (alone or in combination with existing ones) to use against cancer.

## 1. Introduction

Glioblastoma (GBM) is the most common and aggressive subtype of the malignant gliomas, which is characterized by intense proliferation, invasion, and intratumour heterogeneity. The previous classification of gliomas that was generated by the World Health Organization (WHO) organized this tumour type in four classes. WHO grade I corresponds to astrocytic, oligodendroglial, and mixed oligo-astrocytic gliomas, and includes the most common pediatric gliomas. WHO grade II are low-grade gliomas, whereas the most aggressive tumours and high-grade or anaplastic, fast-growing gliomas are designated WHO grades III and IV. In human populations, the most frequent and malignant type is GBM WHO grade IV. In 2016, the WHO shifted to a new classification system based on both phenotype and genotype [1], where the diffuse gliomas include WHO grade II and III astrocytic tumors, grade II and III oligodendrogliomas, and grade IV GBM. In the most aggressive GBMs, mutations in the genes of tumour protein p53 (TP53) and phosphatase and tensin homolog (PTEN) as well as epidermal growth factor receptor (EGFR) amplifications are quite common [1]. 

The description in many tumour types of “biological plasticity”, showing that a single, theoretically homogeneous population of cells may generate phenotypically heterogeneous cells in distinct states of differentiation, and the presence of stem-like markers inside these tumour populations have led to the cancer stem cell (CSC) hypothesis. This hypothesis suggests that the oncogenic capacity and biological plasticity of a tumouris that is maintained by a minor fraction of cells with stem-like properties, denoted as CSCs or tumor-initiating cells (TICs). In some cases, these are detectable by the high level expression of markers, such as hyaluronic acid receptor (CD44), prominin-1 (CD133), Nestin, or SRY (sex-determining region Y)-box 2 (Sox2), are expressed, among others. 

This hypothesis was initially supported by the identification of stem-like cell populations in human leukaemia [2,3], and cells with analogous properties have been described in many solid tumors. In brain tumors, a subpopulation CD133+ cells were characterized as CSCs [4], and when isolated, they exhibited CSC properties in vitro and in vivo [5,6]. The ability for self-renewal as well as the capacity to generate different cell phenotypes provides a suitable explanation for the heterogeneity of CSC subpopulations that are observed within tumours [4]. Additionally, the identification and characterization of highly tumorigenic subpopulations within gliomas has opened the possibility of development of novel anti-glioma therapeutics.

## 2. CSCs/TICs

Cancer cells initiate and drive tumour progression forward, carrying oncogenic and tumour suppressor mutations that define cancer as a genetic disease. Independently of semantic questions about whether they should be denoted as CSCs or TICs, these tumour cell subpopulations possess the ability to self-renew and produce differentiating progeny, much like their normal counterpart. One open question is whether they are derived from normal stem cell populations, or may be generated from a “dedifferentiation” of a more differentiated cell. Indeed, Ronald DePinho’s group demonstrated that the combined loss of tumour uppressor proteins p16 and p19, from the Ink4a/Arf locus, enables mature astrocyte dedifferentiation through EGFR activation. Certainly, the transduction of Ink4a/Arf(^−/−^) neural stem cells (NSCs), or astrocytes with constitutively active EGFR, induced a comparably high-grade glioma phenotype [7,8]. Thus, it has been proposed that oncogene-induced dedifferentiation of mature cells in the brain to a stem/progenitor-like state leads to heterogeneous glioma tumours (for review see [9]). When considering all these data, the initiation of oncogenesis perhaps is not a question of “unique cell type”, but might be a combination of mutations that first generate this stem-like phenotype with a subsequent accumulation of mutation that boost the oncogenic progression. 

The genetically-acquired plasticity of these cells allows progression and maintenance of this aggressive tumour type, and even formation of its own blood vessels by transdifferentiation [10]. The data also supports the view that was originally proposed by Ronald DePinho and his group [7], that the dysregulation of specific genetic pathways, rather than the cell of origin, dictates the emergence and phenotype of high-grade gliomas [8]. For many tumour types, it is generally accepted that when a primary tumour is formed, a CSC subpopulation is generated that may self-renew as well as generate more differentiated derivatives, and that these heterogeneous progeny form and increase the tumour size. However, we still do not have much information about how this process of interconversion occurs in vivo, or the mechanism that regulates the dynamic equilibrium that exists between non-CSCs and CSCs. 

CSCs/TICs can be defined operationally through their ability to efficiently seed new tumours upon inoculation into recipient host mice [11,12]. This functional definition is often complemented by measuring markers that are also expressed by the normal stem cells of the tissue-of-origin [13]. Indeed, in almost all gliomas and tumour cells from neural lineages we have analysed, we obtained and enriched CSC-like cells using defined cell culture media in an anchorage-independent system [6,14,15]. Expression of glioma stem-markers, such as CD133, Nestin, and Sox2 is significantly increased in tumours growing in suspension, as neurospheres [16,17,18]. In fact, CD133+ and CD133− subpopulations that were obtained from primary tumours presented differential gene repertoires and dissimilar capacity to generate new tumours in vivo upon implantation in immunodeficient mice [17,19,20,21,22,23]. CD133 is a membrane protein interconnected with the Phosphoinositide 3-kinase Class I (PI3K)/Thymoma viral oncogene protein kinase (Akt) pathway; phosphorylation of tyrosine-828 within its C-terminal cytoplasmic domain mediates direct interaction with the PI3K 85 kDa regulatory subunit (p85) and favours the preferential activation of the Akt pathway in glioma stem cells relative to matched non-stem cells [24].

Certainly, it is plausible that the phenotypic plasticity working within tumours may produce bidirectional interconversion between CSCs and non-CSCs, resulting in a dynamic variation in the relative abundance of CSCs and explaining the cellular intratumoral heterogeneity. Indeed, we do not have complete evidence showing that in gliomas there is an intrinsically heterogeneous CSC-like population with a gradation of CD133, Nestin, and Sox2, but it appears to be plausible.

Recent research has linked the acquisition of CSC traits with the epithelial-mesenchymal transition (EMT) trans-differentiation program [25,26,27]. Induction of EMT in certain cellular models can bring many of the defining features of “stem cells”, and stem-like cells that were isolated either from mouse or human mammary glands or mammary carcinomas similarly express EMT markers (snail, vimentin and fibronectin) [26]. The EMT program can confer on such cells the self-renewal capability that is crucial to their subsequent clonal expansion [28], and may allow for cancer cells to physically disseminate from primary tumours, a capacity that was developed by many tumour cells. In addition, EMT cells may generate antigenic phenotypes that were associated with both normal and cancer stem cells.

Thus, the EMT program is implicated in tumour plasticity, which can be engaged reversibly from EMT to mesenchymal-epithelial transition (MET) [29]. For example, an EMT can convert epithelial carcinoma cells into mesenchymal, fibroblast-like cancer cells that may well assume the duties of cancer-associated fibroblasts (CAFs) in some tumours [30,31]. Remarkably, several recent reports have documented the ability of glioblastoma cells (or possibly their associated CSC subpopulations) to transdifferentiate into endothelial-like cells that can substitute for *bona fide* host-derived endothelial cells in forming a tumour-associated neovasculature [10,31,32]. 

Tumour heterogeneity has important implications for effective cancer therapies. The heterogeneity and capacity for interconversion among phenotypes make tumours more adaptable not only in different physiological and tissue environments, but also in the resistance to therapy. It has been reported in a variety of tumour types that some cells with CSC properties are more resistant to chemotherapeutic treatments or radiotherapy [25,33,34], which may help to explain the recurrence of many tumour types.

## 3. The “Long Road” to Oncogenesis

In 2011, Hanahan and Weinberg proposed a framework for understanding the significant diversity of neoplastic diseases, using six general hallmarks modified in all cancer types [35]. They postulated that normal cells evolving to a neoplastic state, must acquire a succession of these hallmark capabilities, and that the multistep process of human tumour pathogenesis may be reorganized by the need of incipient cancer cells to become tumorigenic and ultimately malignant. Among these hallmarks, sustained cell survival and proliferation in combination with evasion of apoptotic checkpoints are early capabilities that are enhanced in initially transformed cells to form a tumour. 

From the pioneer data of ras-mediated oncogenic transformation, summarized for instance by M. Malumbres and M. Barbacid [36], the activation by mutation in several elements of the mitogen-activated protein (MAP)-kinase pathway have been described [37]. Similarly, mutations in the phosphoinositide3-kinase (PI3K)-Akt pathway have been detected in many arrays of tumour types [38,39]. Both pathways represent the molecular bases of survival and proliferation in almost all cell types.

The PI3K-Akt pathway is considered to be one of the most relevant pathways that is involved in survival and proliferation, both activated in cancer cells. The involvement of the PI3K-Akt pathway in the development and progression of cancer has been studied extensively [40], establishing Akt1 as an oncogene [41]. Some elements in this pathway may control tumour cell proliferation [42,43], and/or the maintenance of the tumour phenotype [44]. Indeed, Akt is frequently activated in human cancers (reviewed in [45]) and its hyper-activation (directly by over-expression or mutation, or indirectly through alterations to PTEN) offers protection against apoptosis and at least in part promotes cell-cycle progression [46], which are two major hallmarks of cancer [35,47].

## 4. Akt in Cancer

Akt (also known as protein kinase B, PKB) is a widely studied protein that was initially described as the human homolog of a viral oncogene [48], and it belongs to the family of proteins related with protein kinase A, G, and C (AGC family of kinases) [49]. Akt is involved in many biological processes and pathologies, such as metabolism regulation, cell growth, survival, proliferation, cancer, and neurodegenerative disorders [40,50]. In mammals, there are no fewer than three Akt isoforms encoded by three different genes (Akt1/PKBα, Akt2/PKBβ, and Akt3/PKBγ). In addition Akt3 may encode two variants, even though the physiological relevance of such variants is not clear [40,51,52]. These paralogs are closely related and share a high homology at the protein level [50]. A plethora of extracellular signals induce Akt activation through class I PI3K [40]. In this path, the production of phosphatidylinositol (3,4,5)-triphosphate (PIP3) by the lipid kinase leads Akt to translocate to the plasma membrane, where it is activated by phosphorylation through two kinases: phosphoinositide-dependent kinase-1 (PDK1) and mammalian target of rapamycin complex 2 (mTORC2) of two amino acids residues, threonine 308 and serine 473, respectively (amino acid numbers corresponding to the Akt1 isoform) [53]. Furthermore, more putative kinases have been described to phosphorylate, at least threonine 308, such as integrin-linked kinase (ILK) [54]. Finally, active Akt exerts its function through the phosphorylation of a wide range of substrates.

Akt isoforms are differentially expressed and have been related to distinct functions. Akt1 and Akt2 are widely expressed, with especially high levels of Akt2 being present in the heart, skeletal muscle, adipose tissue, and testes, whereas Akt3 expression is mainly restricted to the brain and testes [50]. The generation and analysis of knockout mice for each Akt isoform has also revealed distinct physiological functions: Deletion of Akt1 reduces body and cell size [55,56] Akt2-knockouts show diabetes mellitus-like syndrome [55,57], and Akt3 deletion causes smaller brain size and corpus callosum disorganization [58,59]. Hyper-activation of the PI3K-Akt pathway is involved with progression in the majority of tumour types [60,61]. Moreover, the role of each Akt isoform in tumour development remains unclear despite the fact that each isoform may appear amplified or mutated in different cancer types. For instance, a specific activating mutation of Akt 1 (E17K) is associated with some tumour types [41,62]. In breast cancer, Akt1 appears to play a fundamental role in the propagation of such tumours [60,63,64,65,66], whereas ablation of Akt2 inhibits apoptosis and delays tumour involution [67]. 

In contrast to Akt1, which accelerates the induction of mammary tumours in transgenic mice, Akt2 can promote the metastasis of tumour cells without affecting the latency of tumour development in certain systems [68,69]. We recently analysed the role of Akt isoforms in survival and self-renewal of TICs as well as the correlation between Akt activity and CSC/EMT phenotype. Indeed, we found that Akt plays an important role in cancer and is frequently activated in human tumours (for review see, i.e., [45].

Our first approach used TICs from breast cancer cell lines in an attachment-independent tumour cell growth system with serum-free medium. We demonstrated that the PI3K-Akt pathway includes elements that are essential to maintain the CSC-like phenotype, survival, and EMT characteristics in breast cancer cells and gliomas [70,71]. When we blocked PI3K activity, or when we knocked down Akt (mainly the Akt1 isoform), the survival and population size of TICs (measured as CD44High/CD24Low population) were severely reduced. Unexpectedly, the loss of cell viability provoked a modification of the CSC phenotype, whereby cells expressing stem/mesenchymal characteristics, like CD44High/CD24Low, high Vimentin, and low E-cadherin, were replaced by those with an epithelial-like phenotype (low Vimentin, high E-cadherin). Our data supported the hypothesis that adoption of a CSC phenotype correlated with the EMT phenotype. This strongly suggested that the EMT programme not only may permit cancer cells to disseminate but also might confer on them a self-renewal capacity [26,27]. In addition, we observed that when Akt activity was interfered with or reduced, CSCs appeared to undergo a MET transition before they die. 

This strongly suggests that some Akt-dependent elements are essential in the maintenance of CSC proliferation and phenotype. Next, we wanted to establish whether either Akt1 or Akt2 contributes to a specific aspect of the CSC-like phenotype. Our data showed a prominent role of Akt1, and to a lesser extent, Akt2, in such processes in TICs derived from breast cancer cells. Indeed, shRNA-Akt1 provoked a drastic reduction in the CD44High/CD24Low phenotype, growth capacity, and EMT markers. The reduction of Akt1 in these breast cancer cells induced cell death in which a MET transition preceded apoptosis [71]. In conjunction, these data indicated that the expression of CD44High/CD24Low and other mesenchymal markers in CSC-like cells is tightly linked to TIC survival and that Akt1 may control both. 

Our data are in agreement with some reports using the cell line MCF10A expressing Insulin-like growth factor receptor (IGFR), showing that the down-regulation of Akt1, but not of Akt2, dramatically increased cell migration [68,72,73], which may correlate with Akt1’s role in maintaining the EMT phenotype. 

From these initial data, we cannot rule out the possibility that Akt2 or Akt3 may be more relevant in controlling the CSC phenotype in models from different tumour lineages. Certainly, in our breast cancer model, impairment of Akt1 or Akt2 does not produce identical biochemical profiles, as indicated by the effect on important proteins, such as Survivin or β-catenin [71]. 

At present, the specific role of Akt3 in tumours is not fully understood. In triple-negative breast cancer (TNBC), downregulation of Akt3 significantly inhibits growth in three-dimensional (3D) spheroid cultures and in mouse xenograft models [74]. In glioma, expression of Akt3 mRNA and protein decreases as the malignancy grade increases, in parallel with increased Akt2 mRNA and protein [75]. That study showed that the down-regulation of Akt2 or Akt3, but not Akt1, reduced the phosphorylated form of Bad, resulting in induction of caspase-dependent apoptosis. Similarly, a direct relationship has been reported between human GBM patient outcome and mRNA levels of both Akt1 and Akt2, but an inverse relationship with Akt3 mRNA. Accordingly, Akt3 mRNA levels were higher in less aggressive GBM subtypes, and the overexpression of Akt3 improved survival in a rodent model of GBM [76]. Our data confirm that the U87-MG and U373-MG gliomas express almost negligible amounts of Akt3 when maintained in CSC culture medium. These results open questions about the specific role of each Akt isoform in different tumour types that must be carefully studied. 

More recently, we generated TICs from glioma cell lines or human glioblastomas, which were characterized by the presence of mutant p53 (mp53), and in serum-free medium the cells were enriched with markers, such as CD133 or CD44. The biochemical analysis of mp53 gliomas, growing as CSCs, showed good correlation between high levels of WASP-interacting protein (WIP) and high levels of pAkt and pErk, and also between expression of the stem markers Yes-associated protein 1 (YAP) and Transcriptional co-activator with PDZ-binding motif (TAZ) [77]. We demonstrated that the knockdown of WIP and mp53 both reduced cellular growth and expression of stem markers (CD133, CD44 and YAP/TAZ) in a similar fashion. Next, we checked the capacity of different inhibitors to reduce the levels of these stem markers, and we observed that the PI3K class I inhibitor, GDC-0941, reduced CSC markers, strongly suggesting that elements downstream of PI3K are responsible for maintenance of the TIC phenotype. In order to identify these PI3K-downstream elements, we analysed the different Akt isoforms, among other candidates.

We demonstrated in these TICs that a deficiency of Akt2 strongly reduced CSC-like markers (CD133, CD44, or YAP/TAZ) [71,78], as well as EMT markers, when compared with similar Akt1 knockdown. More interestingly, these Akt2-deficient gliomas were not able to generate brain tumours upon implantation in immunodeficient mice, unlike glioma-derived controls. Moreover, our data demonstrated that this Akt2 function, regulating CSCs and EMT markers, is under the control of mp53, regulating a new pathway of oncogenesis in which WASP-interacting protein (WIP) and YAP/TAZ are downstream elements, which are essential in stemness control. In support of this, when we overexpressed mp53 in human astrocytes in attachment-independent conditions, we obtained CSC-like cells expressing CD133 and EMT markers. These transformed astrocytes used the Akt-WIP-YAP/TAZ path to sustain these TIC properties [78]. 

This new oncogeneic pathway was not specific to cell lineage because similar results were obtained using some breast cancer cell lines with mp53. 

The pathway that is described in these TIC tumour cells was different from those that were described for Akt1 (initially only in some breast cancer cells). In those from Akt1-dependent proliferation, we showed that Foxo and Bim are essential downstream to maintain proliferation and phenotype. In fact, Bim has been associated with apoptosis induced by a lack of adhesion, called “anoikis”, which is very relevant to breast cancer development [79,80]. In the case of Akt2, after experimentally discarding Foxo/Bim, we showed that YAP/TAZ are the main players in maintaining the proliferation and TIC capabilities.

It is important to remember the putative role of β-catenin in the regulation of CSCs and EMT transition. It is generally accepted that this protein plays an essential role in the development and in cancer as a Wnt or Hippo element [77,81]. Furthermore, some authors have described that β-catenin is essential to promote tumorigenic capacity downstream of Akt [24,42,82], as it is a partner of the YAP/TAZ proteins [83]. All these data strongly suggest that β-catenin, directly or indirectly, is a common element of both Akt1 and Akt2 oncogenic signalling 

**Figure 1:**
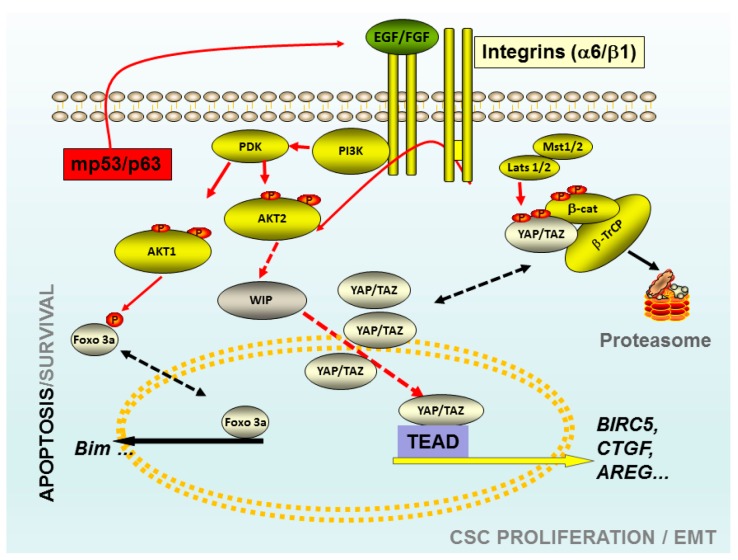
Schematic representation of some elements that play a key role in the tumour-initiating cells (TICs). The Akt 1 isoform may control the viability versus apoptosis in CSCs derived from breast cancer cell lines through the control of FoxO phosphorylation. The knockdown of Akt 1 in these TICs triggered apoptosis through a mechanism FoxO3a-Bim dependent. TICs derived from gliomas, mutations of p53, correlated with high levels of WASP-interacting protein (WIP) protein and Yes-associated protein 1 (YAP)/Transcriptional co-activator with PDZ-binding motif (TAZ). In these TICs the presence of high levels of WIP are essential to maintain the proliferation and CSC-phenotype through mp53 and Akt2. Whereas, high levels of WIP control the high levels of YAP/TAZ, preventing its degradation trough proteasome. Red arrows indicated the pro-oncogenic pathways, black discontinued arrows indicated two potential stages of the same protein; red discontinued arrows indicated not necessarily direct links. Abbreviations: CSC: Cancer Stem cells; PI3K: Phosphoinositide 3-kinase Class I; Akt: viral oncogene protein kinase; Foxo 3a: Forkhead box O3; EGF: Epidermal Growth Factor; FGF: fibroblast growth factor; TEAD: Trancription factor TEA domain member 1; CTGF: Connective tissue growth factor; AREG: Amphiregulin; BIRC5 Baculoviral IAP repeat-containing protein 5; Mst 1/2: Serine/threonine-protein kinase3/4; Lats 1/2: Large tumor suppressor kinase 1/2; β-Trcp: beta-transducin repeat containing protein.

Thus, we can conclude that in the case of Akt1-dependent CSCs, Foxo, and Bim play an important role in cell survival and proliferation [70], whereas in the case of Akt2, the main player is YAP/TAZ signalling [71], directly or indirectly associated with Wnt signalling. Some of the elements that are discussed in this review were schematically represented in the Figure 1. We cannot determine whether β-catenin is a common player interacting with Foxo [84] and the YAP/TAZ element [77].

## 5. Conclusions

In the present work, we have summarized some elements of the PI3K-AKT pathway that are necessary to maintain the CSC-like phenotype as well as their survival, in TICs. Our data and other laboratory data support the working hypothesis that each Akt isoform plays an important and specific role in TIC growth, self-renewal, survival, and EMT phenotype. We cannot discriminate the effects of each Akt isoform on survival, proliferation, and stem-marker generation and maintenance in CSCs. Obviously, the possibility that each isoform may play some specific role in one of these tumour capabilities cannot be ruled out. 

A more complete analysis of how hierarchical signalling is built into oncogenic generation must be done in order to propose/generate new therapeutic approaches (alone or in combination with previous ones) to use against cancer. We still have several open questions, such as how some protein isoforms play specific roles to ensure CSC survival and avoid apoptotic pathways, and how CSC and EMT phenotype and tumour growth are interconnected.
